# Salivary microbial profiles across periodontal severity stage according to the 2018 AAP/EFP classifications: a cross-sectional study

**DOI:** 10.3389/froh.2026.1811009

**Published:** 2026-04-30

**Authors:** Paola E. García-Vázquez, Marco Antonio Pita-Galeana, Mireya Martinez-Garcia, S. Aída Borges-Yáñez, Adriana-Patricia Rodríguez-Hernández, Enrique Hernandez-Lemus

**Affiliations:** 1 Master's and Doctoral Program in Medical, Dental and Health Sciences (PMDCMOS), National Autonomous University of Mexico (UNAM), Mexico City, Mexico; 2 Division of Computational Genomics, National Institute of Genomic Medicine, Mexico City, Mexico; 3 Postgraduate Program in Biochemical Sciences, National Autonomous University of Mexico, Mexico City, Mexico; 4 Department of Dental Public Health, Division of Postgraduate Studies and Research, Faculty of Dentistry, National Autonomous University of Mexico, Mexico City, Mexico; 5 Laboratory of Molecular Genetics, Postgraduate Studies and Research Division, Faculty of Dentistry, National Autonomous University of Mexico, Mexico City, Mexico

**Keywords:** Checkerboard DNA–DNA hybridization, microbial profiles, microbiota, oral dysbiosis, periodontitis, saliva

## Abstract

**Background:**

Periodontitis is a chronic inflammatory disease characterized by progressive destruction of the periodontal supporting tissues and is strongly associated with shifts in the oral microbiota. Saliva has emerged as a promising diagnostic fluid; however, few studies have analyzed its microbial composition at the species level according to the 2018 American Academy of Periodontology/European Federation of Periodontology (AAP/EFP) classification of periodontal diseases.

**Objectives:**

To characterize salivary microbial profiles in a systemically healthy Mexican population across different stages of periodontitis according to the AAP/EFP 2018 classification system, and to evaluate sociodemographic, anthropometric, and clinical characteristics as potential risk factors for periodontitis.

**Methods:**

A cross-sectional study was conducted by analyzing 133 adults classified in four groups: periodontally healthy (*n* *=* *33*), gingivitis (*n* *=* *34*), stage I–II periodontitis (*n* *=* *18*), or stage III–IV periodontitis (*n* *=* *48*). Unstimulated saliva from all participants was sampled and analyzed for 40 bacterial species using Checkerboard, a DNA–DNA hybridization technique. Microbial levels, prevalence, and proportions were compared among groups. Ordinal logistic regression was then used to identify independent predictors of disease severity.

**Results:**

The *Porphyromonas gingivalis* and *Tannerella forsythia* bacteria showed significantly higher levels in advanced periodontitis (*p* *<* *0.001*). Bacteria from the *Orange complex*, such as *Campylobacter rectus* and *Campylobacter showae*, also increased with disease severity. In contrast, commensal species, notably *Gemella morbillorum,* were diminished. Ordinal logistic regression identified age (OR = 1.10, *p* < 0.001), the presence of dental biofilm (OR = 1.03, *p* = 0.005), and higher counts of the *Aggregatibacter actinomycetemcomitans* (OR = 1.19, *p* = 0.005), and *P. gingivalis* (OR = 1.21, *p* < 0.001) microbial species, as positive predictors of periodontitis**.**

**Conclusions:**

The salivary microbiota shows distinct abundance signatures across periodontal severity stages. These patterns are characterized by an enrichment of pathogenic microbial complexes and depletion of commensal species. This aligns with current frameworks placing dysbiosis as a relevant risk factor for periodontitis. These findings also support saliva as a feasible matrix for periodontal screening and risk stratification. This may result particularly useful in resource-limited clinical laboratory settings.

## 1 Introduction

Periodontitis is an oral disease characterized by the progressive destruction of the periodontal supporting tissues and the loss of clinical attachment (1, 2). The condition arises from dysbiosis of the oral microbial community and is closely linked to the accumulation of dental biofilm along tooth surfaces (3, 4). It is also one of the leading causes of tooth loss and may contribute to impaired general health, this is because significant associations have been observed with systemic diseases such as diabetes (5), hypertension (6), cardiovascular disease (7), and Alzheimer's disease (8, 9).

Saliva, the biofluid that coats the entire oral cavity, plays essential roles in chewing, swallowing, and speech (10). It is also an important microbial reservoir. The salivary microbiota consists of bacteria shed from different oral surfaces, including the tongue, palate, mucosa, and teeth (10, 11). Several studies have detected subgingival periodontopathogenic bacteria in the saliva of individuals with periodontitis (10, 12), indicating that saliva reflects bacterial shedding from multiple intraoral sites (13, 14).

Most studies investigating salivary microbiota associated with periodontitis have employed targeted approaches such as quantitative real-time polymerase chain reaction (qPCR)–based methods (15). In addition, sequencing of certain hypervariable regions of the 16S rRNA gene often limits species-level resolution. Although these approaches have been widely used over the past decade, they are constrained either by *a priori* target selection (qPCR) or by insufficient taxonomic resolution (some 16S rRNA regions). Though less comprehensive than next-generation sequencing, the DNA–DNA hybridization, or “Checkerboard” technique, remains a robust tool for focused, species-level microbial comparisons (16–18). Since its original description by Socransky in 1994 using whole genomic probes (17), this technique has been applied to saliva, as well as supragingival and subgingival biofilm samples, to examine microbial composition related to increasing severity and risk factors in dysbiotic conditions such as periodontitis (18–21).

Few studies have applied the 2018 periodontal staging system to salivary microbial profiles, and to date, none have done so in systemically healthy Mexican cohorts using the DNA-DNA hybridization (22–24). Moreover, validation of saliva as a diagnostic matrix represents a significant advance toward non-invasive and accessible tools for the diagnosis and monitoring of periodontitis, particularly in clinical settings with limited resources (25).

Here, we hypothesized that the salivary microbiota exhibits distinct microbial signatures across periodontal severity stages. Such signatures will be characterized by enrichment of pathogenic complexes and depletion of commensal species, reflecting the dysbiotic shift associated with periodontitis.

Therefore, the main goal of the present study is to characterize salivary microbial profiles in a systemically healthy Mexican population across different stages of periodontitis according to the AAP/EFP 2018 classification system, using the Checkerboard DNA–DNA hybridization technique, and to evaluate sociodemographic, anthropometric, and clinical characteristics associated with the severity of periodontitis, such as sex, age, body mass index, oral hygiene index, bleeding on probing, and the decayed, missing, and filled teeth (DMFT) index.

## 2 Materials and methods

### 2.1 Ethics

The study was conducted in accordance with the ethical principles outlined in the Declaration of Helsinki (26) and was approved by the Research and Ethics Committee of the Faculty of Dentistry at the National Autonomous University of Mexico under reference number CIE/0824/03/2024. All participants read and signed an informed consent form prior to enrollment in the study.

### 2.2 Study population

A total of 133 unstimulated saliva samples were collected from adults aged 18 to 60 years. Participants were classified as periodontally healthy, having gingivitis, stage I–II periodontitis, or stage III–IV periodontitis according to the 2018 classification criteria (27). All individuals were recruited from the Division of Graduate Studies and Research at the Faculty of Dentistry, National Autonomous University of Mexico, between June 2024 and September 2025.

No formal *a priori* sample size calculation was performed, as the study was designed as an exploratory cross-sectional analysis that included all eligible participants during the study period. However, a *post hoc* power analysis was conducted under the following assumptions: four groups, Cohen's f effect size of 0.30, *α* = 0.05, k = 4, and *N* = 133. The estimated power for a one-way ANOVA was 0.82 (82%), indicating adequate statistical power given the achieved sample size.

The selection criteria were as follows:

Inclusion criteria
  - Males and females aged 18–60 years (28, 29).
  - Residing in Mexico City or surrounding areas, systemically healthy (with self-reported chronic systemic diseases or conditions).
  - With no oral hygiene performed 24 h before sample collection and without additional use of antiseptic mouthwashes or oral hygiene aids (dental floss, interdental brushes) (30–32).
  - Participants with at least 20 natural teeth, not counting third molars (2, 4, 33–36).
Exclusion criteria
  - Individuals with previous periodontal treatment.
  - Those who had taken antibiotics within the last three months (37, 38).
  - Pregnant or breastfeeding women.
  - Individuals using orthodontic appliances (39–41).

**Figure 1:**
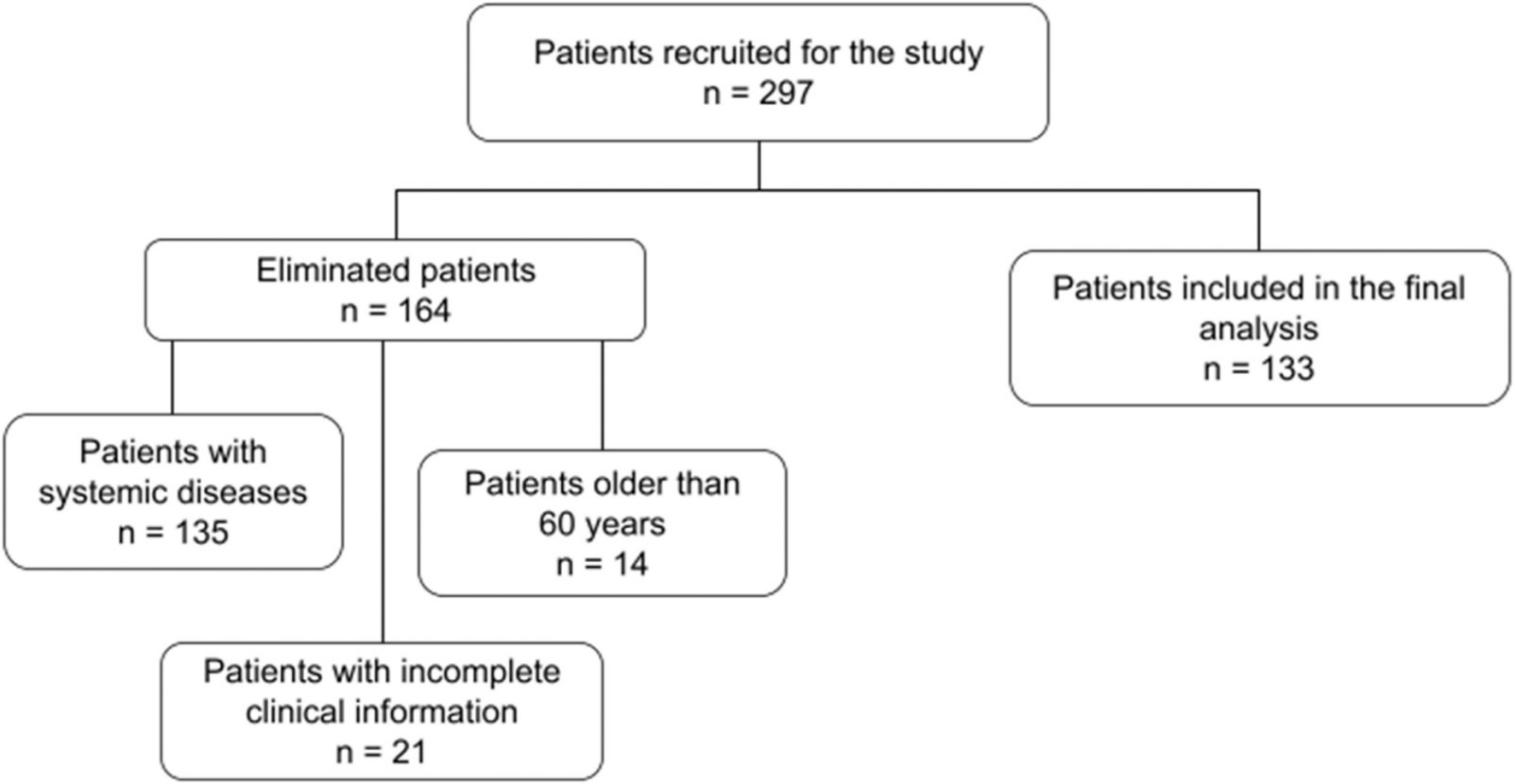
Further elimination of samples that didn't meet inclusion criteria or have incomplete information.

Participants who did not undergo clinical examinations, had incomplete information or contaminated samples or didn’t meet the age and systemic health inclusion criteria were eliminated, as shown in Figure 1.

### 2.3 Sociodemographic data

Sociodemographic information, including sex (defined as sex assigned at birth) and age (recorded in completed years), was collected. Educational level was classified into five categories: complete primary education, middle school, high school, university studies, and postgraduate studies. Current occupation was categorized as homemaker, student, workman, professional, or other. Marital status (defined as the legal status arising from marriage or kinship relationships) was recorded as single or married. Anthropometric parameters, including weight and height, were measured, and body mass index (BMI) was calculated using the formula weight (kg)/height^2^ (m^2^). Current smoking status was recorded as yes or no.

### 2.4 Clinical examination and classification

Clinical examinations were conducted by two calibrated clinicians. Oral hygiene was estimated with a modified Oral Hygiene Index (OHI), which measures the percentage of sites covered by varying levels of dental biofilm and calculus (42). The presence of caries was evaluated using the Decayed, Missing, and Filled Teeth (DMFT) index (43). Bleeding on probing (BoP) was assessed using the Van Weijden bleeding index. BoP was recorded on a per-tooth basis, considering each tooth present as positive when bleeding was observed after probing. The BoP percentage was calculated as the number of teeth with bleeding divided by the total number of teeth examined × 100 (44). Periodontal measurements were systematically performed at six sites per tooth on all natural teeth, excluding third molars. These measurements included probing depth and clinical attachment level. Probing depth (the distance from the gingival margin to the bottom of the sulcus or periodontal pocket), and clinical attachment level [the distance from the gingival margin to the cementoenamel junction (CEJ)]. A full-mouth radiographic series (14 radiographs) was obtained using a digital sensor and a portable x-ray device to support periodontal diagnosis and classification (2, 45).

The PCP11.5 periodontal probe was used for OHI and BoP assessments, whereas the PCPUNC 15 probe was used for periodontal measurements. Examiner calibration was performed prior to the study, achieving Cohen's kappa values greater than 0.7 for periodontal measurements and greater than 0.8 for the remaining indices. Recalibration sessions were conducted every six months to ensure ongoing validity and reproducibility.

### 2.5 Periodontal classification

Participants were classified according to the AAP/EFP 2018 classification system of Periodontal and Peri-Implant Diseases and Conditions as follows:

- Periodontal Health Clinical attachment level (CAL) ≤ 3 mm or radiographical bone loss (RBL) and probing depths (PD) ≤ 3 mm, assuming no pseudo pockets (46)
- Gingivitis. CAL ≤ 3 mm nor RBL and BoP at > 10% of the teeth. Including localized and generalized gingivitis (47).
- Periodontitis in stages I or II. Following the criteria for severity, interdental clinical attachment loss of 1–2 mm (stage I) or 3–4 mm (stage II) and RBL affecting only the coronal third of the root (< 15% for stage I and 15–33% for stage II) (2).
- Periodontitis in stages III or IV. Following the criteria for severity, interdental clinical attachment loss ≥ 5 mm and RBL extending to middle or apical third of the root. There should be evidence of tooth loss ≤ 4 teeth due to periodontal reasons in stage III, and ≥ 5 teeth in stage IV (2).

### 2.6 Saliva samples

Saliva samples were collected during the morning, between 8:00 and 11:00 a.m. to minimize circadian variations in saliva viscosity. Participants were instructed to refrain from performing any oral hygiene procedures for 24 h prior to sample collection and to observe a 6-hour fasting period. A total of 3 mL of unstimulated saliva was collected using the spitting method into pre-labeled 15 mL Falcon tubes and immediately placed on ice. Saliva was aliquoted into 1 mL fractions and centrifuged for 10 min at 10,000 rpm. The supernatant was discarded, and the pellets were resuspended in 150 µL of NaOH to lyse cells and preserve DNA (48).

### 2.7 Microbial evaluation

Whole genomic DNA probes labeled with digoxigenin were prepared using a random primer technique (49). Samples were individually processed for the detection and quantification of 40 microbial species using the DNA–DNA hybridization (“Checkerboard” technique) (17), following previously described protocols (36). The selected bacterial species are representative of subgingival colonization and have been extensively studied in both Mexican and other populations with characterized subgingival microbial profiles (35). This panel includes early colonizers (50), bridge (intermediate) colonizers, and late colonizers, as well as species associated with periodontal health, putative pathogens, and established periodontal pathogens (35, 51, 52). The list of bacterial strains used for probe development can be found in Supplementary Table S1.

DNA was isolated and purified (53) from lyophilized bacterial stocks obtained from the American Type Culture Collection (ATCC, Rockville, MD). Probe specificity and sensitivity were evaluated following the supplier's standardized protocols, and assay sensitivity was calibrated to detect from 10⁴ to 10⁷ cells of each bacterial species.

### 2.8 Statistical analysis

Data was analyzed using the R statistical programming language (version 4.5.2., R Foundation for Statistical Computing, Vienna, Austria). Descriptive statistics were used to summarize sociodemographic and clinical features of the study population (*N* = 133). Categorical variables, including sex, educational level, marital status, and occupation, were reported as frequencies and percentages, while numerical variables (age, microbial abundances and counts) were summarized using means and standard deviations. Shapiro–Wilk tests were applied to assess normality of continuous variables.

Bivariate analyses were used to compare clinical, sociodemographic, and microbiological variables across periodontal status groups. Comparisons between categorical variables were performed using Fisher's exact test, whereas continuous variables and microbial data were analyzed using the Kruskal–Wallis test.

Microbial data include the relative abundances of the 40 target species detected in saliva samples. Salivary microbial compositions were compared across periodontal stages. These contrasts were expressed using mean DNA probe counts ± standard error of the mean (SEM), prevalence (% of positive saliva samples) ± SEM, and proportional representation (% of total DNA probe counts) ± SEM for each species. Microbial complexes were further evaluated by grouping species according to previously described subgingival biofilm complexes. Abundance analyses were adjusted for multiple comparisons, and *post hoc* correction was performed using the Benjamini–Hochberg procedure.

A multivariate ordinal logistic regression model (54) was used to evaluate the association between periodontitis’ severity and sociodemographic, clinical, and microbiological variables. Ordinal logistic regression models can be interpreted as latent variable models, in which the observed categorical outcome is a result of an underlying continuous process, as is the case of the gradual progression of periodontal disease (55). Our sample subgroups were classified into four ordered categories: 1) periodontal health, 2) gingivitis, 3) stage I–II periodontitis, and 4) stage III–IV periodontitis. Potential confounding variables were evaluated and included in the analysis based on biological plausibility and previous literature. These variables comprised age, sex, smoking status, body mass index (BMI), oral hygiene, and socioeconomic indicators. Their inclusion aimed to account for factors that could be associated with both salivary microbial profiles and periodontal severity, thereby reducing the risk of residual confounding in the multivariable models. To reduce potential bias, confounding variables were evaluated and included based on biological plausibility and previous literature, and examiner calibration and standardized clinical procedures were implemented to improve measurement consistency. Odds ratios (ORs), 95% confidence intervals (95% CI) and association *p*-values were calculated. Multicollinearity among the explanatory variables was assessed prior to model fitting. Variable selection for the final model was guided by biological relevance to the disease and was further refined using a linear stepwise approach based on the Akaike Information Criterion (AIC), with the model exhibiting the lowest AIC selected as the most parsimonious. Variable selection can modify the correlation structure among predictors, in this sense, multicollinearity was re-examined in the final model. Variables retained in the final model included age, marital status, DMFT index, BMI, and selected bacterial species (*Aggregatibacter actinomycetemcomitans, Campylobacter showae, Capnocytophaga ochracea, Cutibacterium acnes, Fusobacterium nucleatum sensu stricto, Porphyromonas endodontalis, Porphyromonas gingivalis, and Schaalia odontolytica*). Finally, the proportional odds assumption was evaluated using the Brant test (56) to assess whether the variable's effect remained constant across the periodontal health subgroups.

## 3 Results

From the 133 participants included in the study (Table 1), participants were distributed as follows: 33 with periodontal health, 34 with gingivitis, 18 diagnosed with stage I–II periodontitis, and 48 diagnosed with stage III–IV periodontitis. The participants’ age was 39.2 ± 13.0 years; 58.6% were women, with no significant differences in sex, marital status, educational level, or smoking status, observed among periodontal groups. In contrast, significant differences were observed across periodontal stages for age (*p* < 0.001), occupation (*p* = 0.008), and BMI (*p* = 0.039).

**Table 1:** Sociodemographic and clinical characteristics of the studied population and by periodontal condition and disease.

Variables	Population	Periodontal Health	Gingivitis	Stage I-II	Stage III-IV	*p*
	(*n* = 133)	(*n* = 33)	(*n* = 34)	(*n* = 18)	(*n* = 48)	
Age(*μ*±sd)	38.2 ± 13.0	30.8 ± 10.8	39.6 ± 13.4	31.2 ± 10.8	45.2 ± 10.9	*<0*.*001*
Sex						*0*.574*
Male (*n* (%))	55 (41.3)	13 (39.3)	16 (47.0)	5 (27.7)	21 (43.7)	
Female (*n* (%))	78 (58.6)	20 (60.7)	18 (53.0)	13 (72.3)	27 (56.2)	
Marital status						*0*.078*
Single (*n* (%))	86 (64.6)	26 (78.8)	24 (70.5)	11 (61.1)	25 (52.0)	
Married (*n* (%))	47 (35.3)	7 (22.0)	10 (29.5)	7 (38.9)	23 (48.0)	
Education						*0*.424*
Complete primary (*n* (%))	8 (6.0)	0 (0)	5 (14.7)	0 (0)	3 (6.2)	
Middle school (*n* (%))	25 (18.8)	4 (12.1)	9 (26.4)	4 (22.2)	8 (16.6)	
High school (*n* (%))	14 (10.5)	3 (9.1)	3 (8.8)	3 (16.7)	5 (10.4)	
University (*n* (%))	69 (51.8)	22 (66.6)	14 (41.2)	9 (50.0)	24 (50.0)	
Postgraduate (*n* (%))	17 (12.7)	4 (12.2)	3 (8.9)	2 (11.1)	8 (16.6)	
Occupation						*0*.008*
Homework (*n* (%))	14 (12.3)	2 (6.0)	4 (11.8)	1 (5.5)	7 (14.95)	
Student (*n* (%))	26 (18.8)	11 (33.3)	6 (17.7)	6 (33.4)	3 (6.2)	
Workman (*n* (%))	16 (11.5)	1 (3.0)	2 (5.8)	3 (16.7)	10 (20.8)	
Professional (*n* (%))	57 (41.3)	18 (54.5)	13 (38.3)	7 (38.9)	19 (39.5)	
Other (*n* (%))	20 (15.9)	1 (3.3)	9 (26.4)	1 (5.5)	9 (18.7)	
Smoking						*0*.*908*
No	111 (83.5)	28 (84.8)	29 (85.3)	14 (77.8)	40 (83.3)	
Yes	22 (16.5)	5 (15.2)	5 (14.7)	4 (22.2)	8 (16.7)	
Clinical variables						
BMI (μ±sd)	27.7 ± 13.3	25.2 ± 4.6	25.8 ± 4.1	25.7 ± 3.9	31.6 ± 20.9	0.039*
CAL (μ±sd)	4.4 ± 2.3	2.9 ± 0.3	2.9 ± 0.3	4 ± 0	6.4 ± 2.6	*<0*.*001*
RBL (μ±sd)	18.5 ± 6.8	14.9 ± 2.7	17.4 ± 5.9	16.6 ± 2.6	22.7 ± 8.3	*<0*.*001*
PD (μ±sd)	4.1 ± 1.3	3.0 ± 0.4	3.4 ± 0.6	4 ± 0	5.3 ± 1.4	*<0*.*001*
BoP (μ±sd)	25.0 ± 21.4	3.0 ± 3.4	27.8 ± 15.8	30.1 ± 20.8	36.2 ± 21.5	*<0*.*001*
Surfaces with biofilm greater than 2/3 (μ±sd)	27.4 ± 22.7%	14.3 ± 14.6%	22.1 ± 18.7%	31.6 ± 21.3%	38.7 ± 24.7%	*<0*.*001*
DMFT (μ±sd)	9.7 ± 5.3	9.2 ± 5.3	9.9 ± 6.0	8.3 ± 5.6	10.6 ± 4.6	*0*.*417*
Decay (μ±sd)	2.3 ± 2.3	1.3 ± 1.4	2.5 ± 2.6	2.1 ± 2.4	3.0 ± 2.5	*0*.*013*
Filled (μ±sd)	6.7 ± 5.1	7.6 ± 5.1	6.3 ± 5.3	5.7 ± 5.7	6.6 ± 4.7	*0*.*542*
Missing teeth (μ±sd)	0.6 ± 1.4	0.8 ± 1.5	0.6 ± 1.2	0.2 ± 0.6	0.7 ± 1.6	*0*.*531*
Without decay (μ±sd)	15.9 ± 5.6	16.7 ± 5.1	14.7 ± 6.2	18.2 ± 6.4	15.3 ± 5.1	*0*.*174*

BMI, body mass index; CAL, clinical attachment level; RBL, radiographic bone loss; PD, pocket deep; BoP, bleeding on probing; DMFT, decayed, missing, and filled teeth; n, number of patients; µ, mean; sd, standard deviation; p, *p*-value associated with Kruskal–Wallis test.

* *p*-value associated with Fisher's exact test.

**Figure 2:**
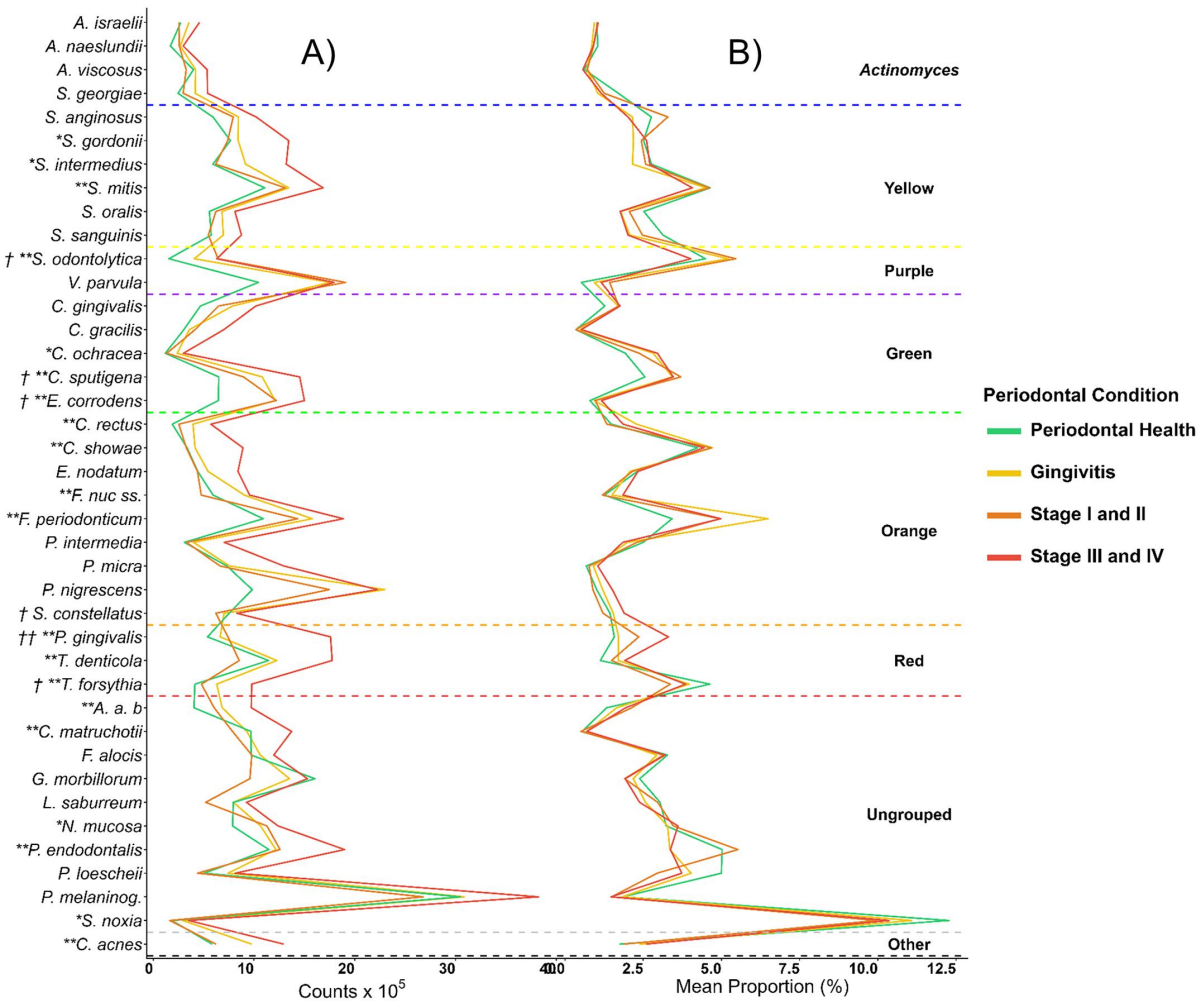
**(A)** Mean total individual levels (total DNA probe counts×10^5^) and **(B)** mean proportion (% total DNA probe count) of 40 individual bacterial species across the 136 participants of the study diagnosed with periodontal health, gingivitis, stages I and II and stages III and IV periodontitis. *A. a.b.: Aggregatibacter actinomycetemcomitas* stp. b. *P. melaninog*.: *Prevotella melaninogenica*. Differences of mean total levels (* *p* *<* *0.05*) and Mean proportions (*† p* *<* *0.05*) between periodontal conditions were determined using Kruskal–Wallis and were further corrected.

Checkerboard bacterial profiles revealed different signatures across periodontal conditions (Figure 2A). Species from the Red complex showed a consistent increase with disease severity. *P. gingivalis* counts were significantly higher mean in stage III–IV periodontitis compared with the periodontal health and gingivitis groups (17.6 × 10⁵ *vs*. 5.4 × 10⁵; *p* < 0.001). *Tannerella forsythia* also increased significantly in advanced disease (9.7 × 10⁵ *vs*. 4.1 × 10⁵; *p* = 0.001).

Bacteria from the Orange complex, especially *Campylobacter rectus* and *C. showae*, displayed significantly higher counts in the stage III–IV group (*p* = 0.001 and *p* = 0.007, respectively). Similarly, *Eikenella corrodens* and *Fusobacterium periodonticum* exhibited significantly higher levels in stage III–IV periodontitis (*p* < 0.001 and *p* = 0.011, respectively) (Figure 2A and Supplementary Table S2).

In contrast, bacteria commonly associated with periodontal health showed reduced levels with clinical severity. *Gemella morbillorum* for example, was decreased in the gingivitis, stage I–II, and stage III–IV groups; however, such differences did not reach statistical significance (Supplementary Table S2).

Regarding the mean proportional representation of species (Figure 2B and Supplementary Table S3), *red complex* bacteria accounted for a significantly larger fraction of the total salivary microbiota in individuals with periodontitis compared with those with periodontal health or gingivitis. Conversely, commensal species such as *Streptococcus mitis* and *Streptococcus sanguinis* predominated in periodontal health but were reduced in disease groups.

**Figure 3:**
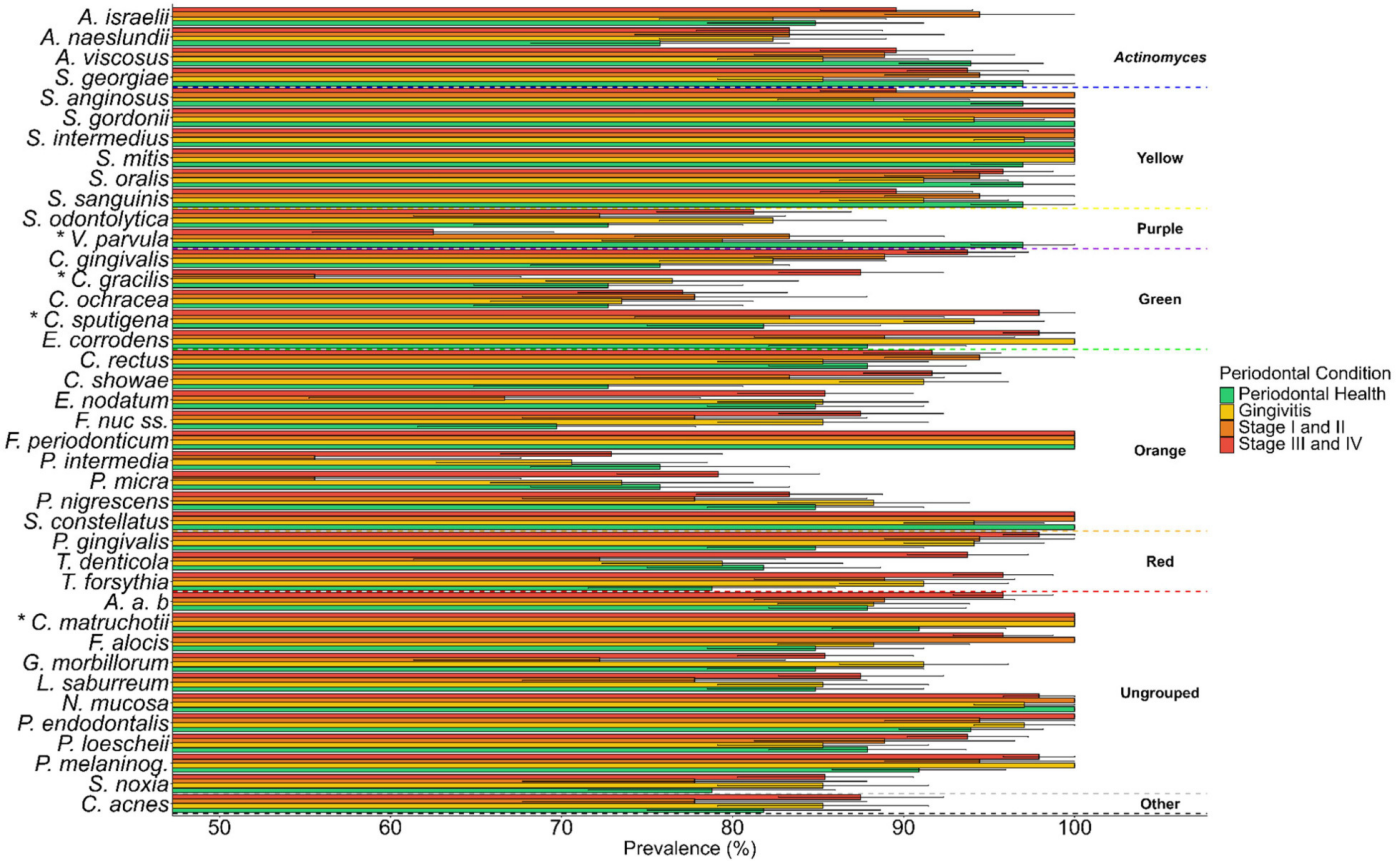
Prevalence of bacterial species in each periodontal condition ± standard error through the checkerboard DNA - DNA hybridization technique. *A. a.b.: Aggregatibacter actinomycetemcomitas* stp. b. *P. melaninog*.: *Prevotella melaninogenica*. Differences of prevalence between groups was determined using Kruskal–Wallis with *post hoc* multiple comparison correction was performed with the Benjamini–Hochberg procedure: * *p* *<* 0.00125*.*

Prevalence analyses (Figure 3 and Supplementary Table S4) reflected similar patterns. The detection frequency of *P. gingivalis* and *T. forsythia* increased with disease severity, whereas *Veillonella parvula* and *Actinomyces viscosus* showed reduced prevalence in the stages III–IV periodontitis group.

**Figure 4:**
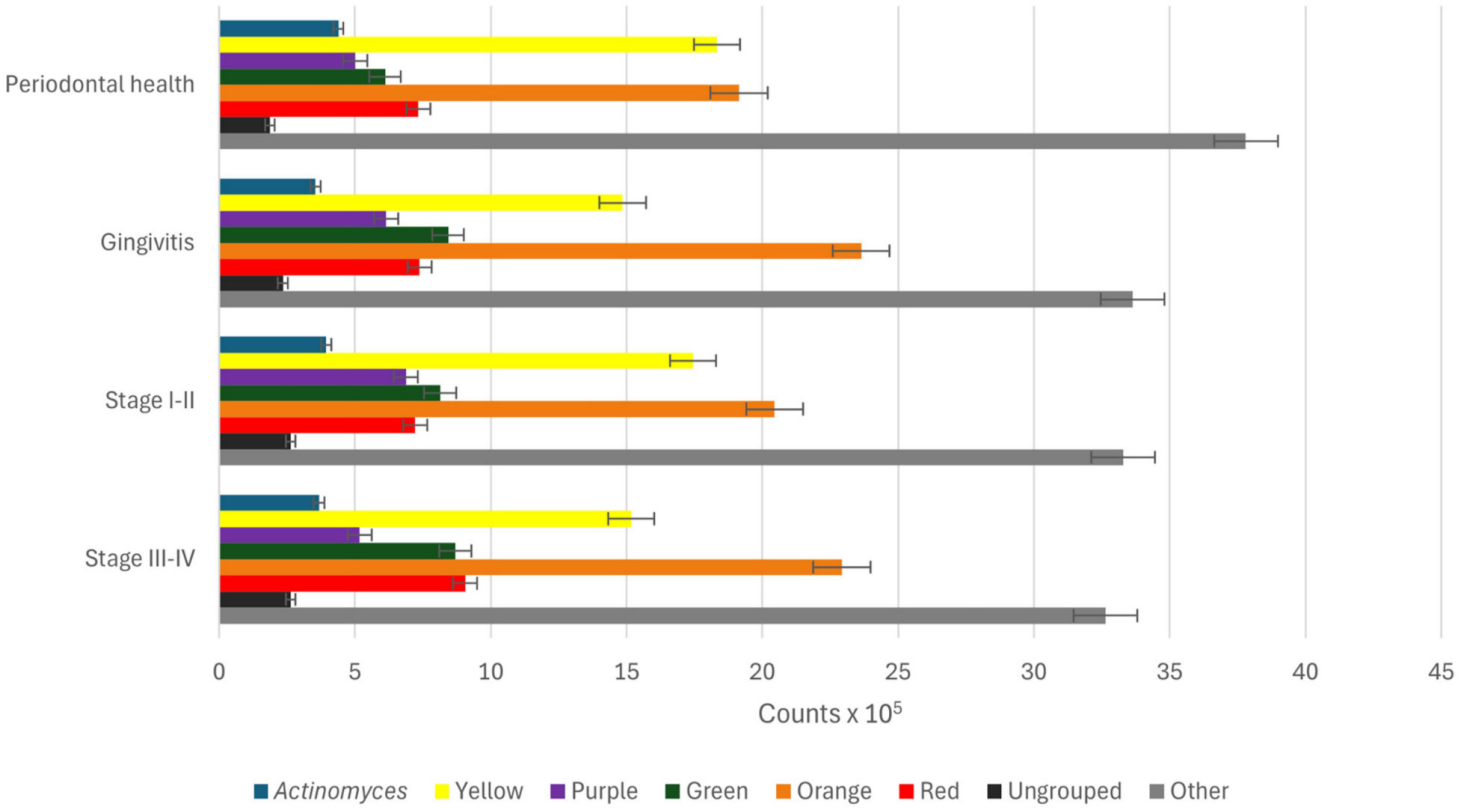
Distribution of the bacterial complexes identified through the checkerboard DNA - DNA hybridization technique according to periodontal condition: periodontal health, gingivitis, periodontitis in stages I and II and periodontitis in stages III and IV. Each circle represents the relative proportions of classical bacterial complexes (Yellow, Purple, Green, Orange and Red) as well as members of the *Actinomyces* genus (dark blue), the Ungrouped (light blue) and other bacterial species grouped as Others (grey).

Mean proportions analysis of bacterial complexes revealed characteristic distribution patterns across periodontal conditions (Figure 2B and Supplementary Table S5). In individuals with periodontal health (Figure 4A), bacteria from the “Other” complex represented the largest proportion of the salivary microbiota (37.8%), followed by those of the Orange (19.1%) and Yellow (18.3%) complexes. In gingivitis (Figure 4B), the overall distribution was very similar. Nevertheless, an increase in microbes from the Orange complex (23.6%) and a slight elevation in those from the Red complex (7.4%) were observed. The ones from the “Other” complex remained relatively abundant (33.6%), whereas the Yellow complex was reduced when compared with periodontal health (14.9%).

For stages I–II periodontitis (Figure 4C), a progressive increase in the proportions of the Orange (20.5%) and Red (7.2%) complexes, accompanied by a decline in health-associated commensal groups, including *Actinomyces* (3.9%) and the Green complex (8.1%) become evident. The “Other” complex remained, however, the most abundant (33.3%). For stage III–IV periodontitis (Figure 4D), the Orange complex reached one of its highest proportions (22.4%), and the Red complex was over-represented compared with the other groups (9.1%). In contrast, the Yellow complex was reduced in relation to periodontal health (15.2% *vs*. 18.3%). Additionally, the *Actinomyces* (3.7%) and Green (8.7%) complexes—both associated with periodontal health—showed lower proportions in the most advanced disease stages.

Multicollinearity of independent variables was assessed using Variance Inflation Factor (VIF). Evidence of multicollinearity was observed among sociodemographic, clinical and microbiological variables (Supplementary Table S6), but was removed after variable selection (Supplementary Table S7). The final multivariate ordinal logistic regression model (Table 2) identified several independent factors associated with periodontitis severity. Increasing age (OR = 1.10, 95% CI: 1.05–1.15), the presence of dental biofilm covering more than two-thirds of tooth surfaces (OR = 1.03, 95% CI: 1.00–1.04), *S. odontolytica* (OR = 1.14, 95% CI: 1.05–1.22), *A. actinomycetemcomitans* (OR = 1.19, 95% CI: 1.05–1.34), and *P. gingivalis* (OR = 1.21, 95% CI: 1.05–1.22) were positively associated with disease severity. In contrast, each unit increase in the DMFT index was associated with a 10% reduction in the odds of periodontitis (OR = 0.90, 95% CI: 0.82–0.97). Similarly, *C. ochracea* (OR = 0.44, 95% CI: 0.48–0.88), *F. nucleatum sensu stricto* (OR = 0.88, 95% CI: 0.82–0.94), and *P. endodontalis* (OR = 0.91, 95% CI: 0.86–0.96) were associated with a modest reduction in the likelihood of periodontitis. Smoking status was included in the model; however, despite an OR of 1.15 (95% CI: 0.44–2.94), it did not reach statistical significance (*p* = 0.776). The proportional odds assumption for this model was satisfied according to the Brant test (Supplementary Table S8).

**Table 2:** Ordinal logistic regression model of periodontitis severity adjusted for sociodemographic, clinical, and microbiological variables.

Variables	OR	(95% CI)	*p*
Age	1.10	(1.05–1.15)	< 0.001
Marital status	0.33	(0.12–0.91)	0.033
Smoking	1.15	(0.44–2.94)	0.776
Surfaces with biofilm greater than 2/3	1.03	(1.00–1.04)	0.005
DMFT	0.90	(0.82–0.97)	0.012
BMI	1.08	(0.99–1.18)	0.074
*A. actinomycetemcomitans*	1.19	(1.05–1.34)	0.005
*C. acnes*	1.07	(1.00–1-13)	0.023
*C. ochracea*	0.66	(0.48–0.88)	0.005
*C. showae*	1.08	(0.97–1.19)	0.126
*F. nucleatum sensu stricto*	0.88	(0.82–0.94)	< 0.001
*P. endodontalis*	0.91	(0.86–0.96)	0.001
*P. gingivalis*	1.21	(1.09–1.33)	< 0.001
*S. odontolytica*	1.14	(1.05–1.22)	0.001

Additionally, a secondary model including only the active dental caries component of the DMFT index was evaluated; however, no association was observed between periodontal severity and dental caries (OR = 0.990, 95% CI: 0.84−1.17) (Supplementary Table S9).

**Figure 5:**
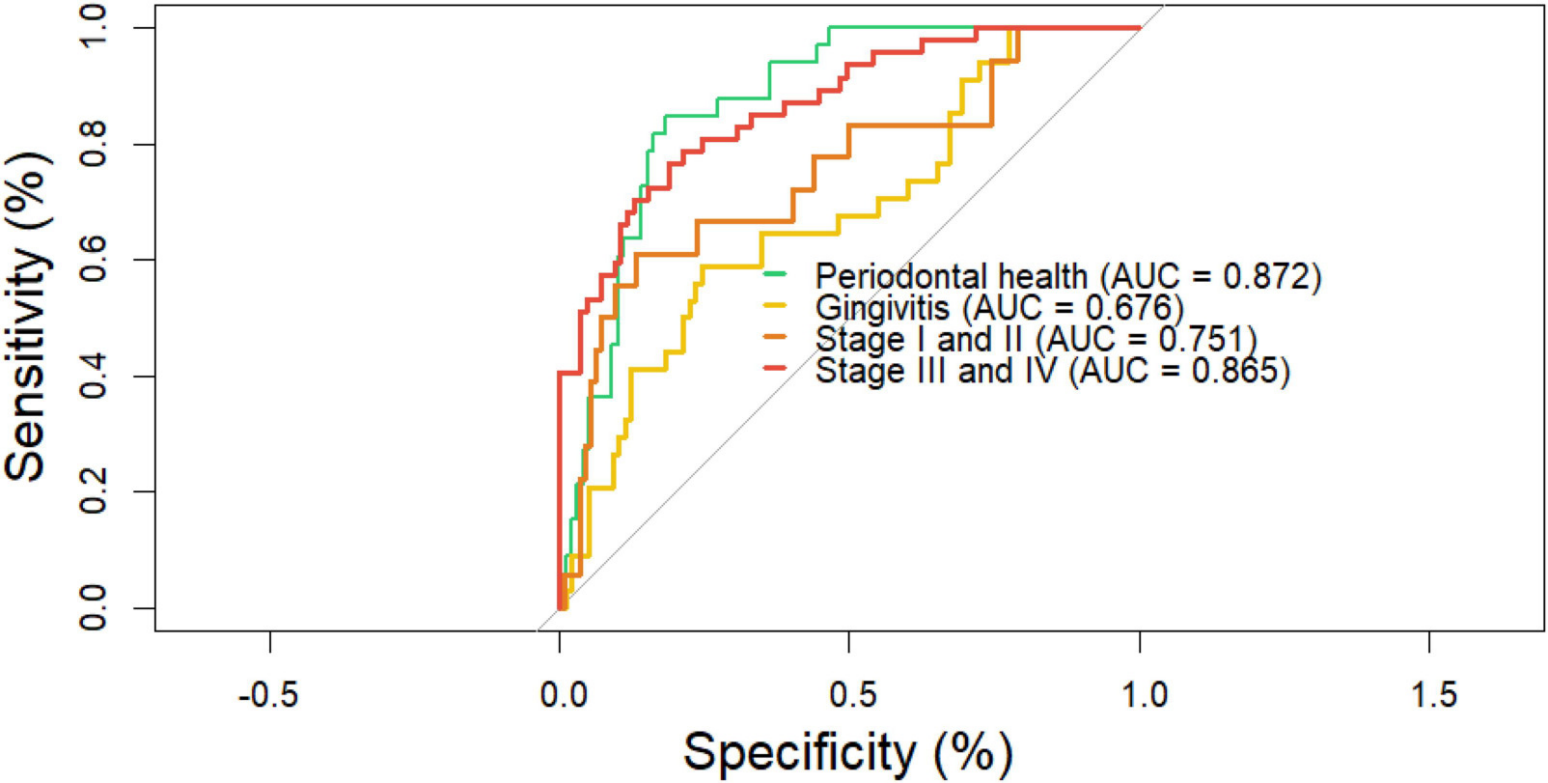
Receiver operating characteristic (ROC) curves of the ordinal logistic regression model including eight bacterial species and five sociodemographic variables. The model achieved high discriminatory capacity for periodontal health (AUC = 0.872; 95% CI: 0.81–0.93; *p* *<* *2.2* *×* *10−^1^⁶*) and Stages III–IV periodontitis (AUC = 0.865; 95% CI: 0.80–0.92; *p* *<* *2.2* *×* *10−^1^⁶*), whereas discrimination was lower for gingivitis (AUC = 0.676; 95% CI: 0.57–0.78; *p* *<* 0.001) and Stage I–II periodontitis (AUC = 0.751; 95% CI: 0.61–0.88; *p* *<* 0.003). *p*-value derived from DeLong's test against the null hypothesis of AUC = 0.5.

The ordinal logistic regression model developed to discriminate among periodontal conditions incorporated eight bacterial species and five sociodemographic variables. Discriminatory performance was evaluated using receiver operating characteristic (ROC) curve analysis implemented in the *pROC* R package (57). Area under the curve (AUC) values with 95% confidence intervals were reported for each periodontal category. Statistical significance was set at *p* < 0.05. The model demonstrated excellent discrimination for periodontal health (AUC = 0.872; 95% CI: 0.81–0.93; *p* < 2.2 × 10⁻^16^) and for stages III–IV periodontitis (AUC = 0.865; 95% CI: 0.80–0.92; *p* < 2.2 × 10⁻^16^). In contrast, discriminatory performance was modest for gingivitis (AUC = 0.676; 95% CI: 0.57–0.78; *p* < 0.001) and for stages I–II periodontitis (AUC = 0.751; 95% CI: 0.61–0.88; *p* = 0.003) (Figure 5).

Taken together, these findings indicate that the salivary microbiota varies significantly according to periodontal condition, with enrichment of pathogenic red and orange complex species in advanced disease stages and depletion of commensal taxa associated with periodontal health.

## 4 Discussion

The aim of the present study was to characterize salivary microbial profiles in a systemically healthy Mexican population across different stages of periodontitis, according to the current Classification of Periodontal and Peri-Implant Diseases and Conditions proposed by the AAP/EFP in 2018. Our findings revealed characteristic signatures of microbial dysbiosis associated with periodontal disease severity, a result consistent with previous studies of the salivary microbiota (23, 58, 59). Saliva sampling is relatively easy to perform, non-invasive, and does not require specialized training for collection. Hence, it represents a practical diagnostic matrix for oral microbiota research and a useful adjunct to clinical periodontal diagnosis, prognosis, and disease monitoring.

Our results showed a gradual increase in both the levels and prevalence of *red complex* species (*P. gingivalis* and *T. forsythia*) in relation to increasing clinical severity of periodontitis. This trend has been reported in other populations using subgingival microbiota samples (60, 61) and has also been observed in previous studies conducted in Mexican populations (62, 63). Moreover, among the bacterial variables included in the regression model, *P. gingivalis* and *C. rectus* were identified as positive predictors of periodontitis. The model achieved an AUC of 86.8%, with 80.8% sensitivity and 80.0% specificity for stages III–IV periodontitis, and an AUC of 89.0%, with 93.0% sensitivity and 74.0% specificity for periodontal health, demonstrating robust discrimination between health and disease states.

These findings are consistent with the bivariate analyses and with previous investigations employing polymerase chain reaction (PCR) (64) and next-generation sequencing (NGS) (23, 65, 66) approaches, which have identified members of the red and orange complexes as key pathogens in increased clinical severity of periodontitis (67). *P. gingivalis*, in particular, has been previously shown (in experimental models) to promote biofilm maturation (68), sustain dysbiosis, attenuate antimicrobial responses (69), and potentially subvert host immune mechanisms (70). Furthermore, it has been discussed that its ecological predominance in saliva may reflect continuous shedding from subgingival biofilms, highlighting its potential as a salivary biomarker for periodontal disease. Collectively, our results suggest that microbial dysbiosis is not confined to the subgingival niche but can indeed be detected in the salivary microbiota (71), a finding of clinical relevance given that salivary sampling is less invasive than subgingival sampling (72).

We also found that orange complex species such as *Fusobacterium nucleatum sensu stricto* and *Prevotella intermedia* were commonly increased in individuals with periodontal disease, supporting their role as secondary colonizers that facilitate adhesion and co-aggregation of more periodontopathogenic species (73). In contrast, bacteria considered commensal or associated with periodontal health, including *S. sanguinis*, *Actinomyces naeslundii*, and *G. morbillorum*, showed a marked decrease in both proportional abundance and prevalence in periodontitis groups (74), indicating a shift toward dysbiosis and the establishment of a more pathogenic oral ecosystem (73). Moreover, species such as *C. ochracea* (Green complex) and *F. nucleatum sensu stricto* (Orange complex) exhibited odds ratios below 1. This finding is noteworthy, as it contrasts with the well-established role of the putative periodontal pathogen as a key facilitator of late recognized periodontal pathogen colonization (75).

An important observation in this study is that although the overall proportions of the Yellow and Green complexes decreased in periodontitis compared with periodontal health, some individual Yellow complex species (such as *Streptococcus oralis* and *S. gordonii*) were present at higher proportions and levels in the most severe stages of periodontitis (stages III and IV) (76). This pattern may reflect secondary recolonization following periodontal tissue destruction and expansion of ecological niches (77), whereby certain commensal bacteria re-establish themselves in the oral cavity without conferring the same protective effects observed under healthy conditions (78). Furthermore, bacteria traditionally regarded as health-associated may actually adapt to inflamed environments and actively participate in dysbiotic biofilms (79), thereby shifting from commensals to symbiotic bacteria. For example, *S. gordonii* has been associated to heme acquisition, an essential iron source that supports the growth and proliferation of *P. gingivalis* (80, 81).

Additional factors positively associated with periodontitis in the ordinal logistic regression analysis included increasing age (OR = 1.10; *p* < 0.001) and the presence of dental biofilm covering more than two-thirds of tooth surfaces (OR = 1.03; *p* = 0.005). These findings are consistent with previous reports describing periodontitis as a chronic disease that progresses over time (82, 83), with the likelihood of advanced stages increasing with age (84). This association aligns with population-level evidence demonstrating higher salivary bacterial loads and inflammatory markers in older individuals, as well as increased counts of periodontopathogens in those with generalized bone loss, suggesting that age-related changes in the salivary milieu may partially mediate the observed age effect beyond periodontal status alone (85). Additionally, periodontitis is closely linked to dental biofilm accumulation (82, 86), as this ecological niche supports the proliferation of bacteria that drive early inflammatory responses in susceptible hosts (82, 87).

BMI showed a positive odds ratio; however, it did not reach statistical significance in the model (OR = 1.08; *p* = 0.074). Interestingly, even when several studies have reported an association between higher BMI and increased severity of periodontitis (88–91), others have shown that this relationship diminishes or becomes non-significant after adjustment for confounding variables such as age and oral hygiene habits (92). Larger studies amenable to covariate and confounder analysis are needed to firmly establish this effect.

The ordinal regression analysis also indicated that for each unit increase in the DMFT index, the odds of periodontitis decreased by approximately 10%. In other words, a higher number of decayed, missing, and filled teeth was associated with a lower likelihood of periodontitis. It is worth mentioning that missing teeth (one of the components of DMFT) may be a confounding factor in this association. However, in our case the average number of missing teeth in our study is 1, and thus missing teeth is an unlikely confounder for this cohort. On the other hand, although both dental caries and periodontitis are endogenous infections influenced by shared socio-behavioral factors (93), the most plausible explanation for this inverse association is their distinct microbial etiologies, which should be considered largely independent (94). Despite sharing environmental conditions such as biofilm accumulation, substrate availability, and poor oral hygiene, the primary microorganisms involved in caries and periodontitis differ substantially (95).

The use of saliva samples instead of subgingival biofilm represents a logistical and clinical advantage, as saliva collection is non-invasive and easier to standardize. Our findings are in agreement with previous studies suggesting that salivary analysis can reflect the overall microbial composition of the oral environment and effectively differentiate between periodontal health and diseased states (12, 96). Nevertheless, saliva represents a composite of multiple oral ecological ecosystems, and its interpretation should therefore be properly contextualized (22).

By identifying specific microbial signatures associated with different stages of periodontitis, our results support the potential development of population-level screening strategies, particularly in resource-limited clinical laboratory settings and periodontal epidemiological studies, where comprehensive subgingival biofilm analysis across large populations may be impractical or prohibitively expensive (25). Furthermore, stratification of microbial patterns by disease stage may contribute to therapeutic monitoring by providing a non-invasive means of assessing treatment outcomes over time (12, 97), especially when integrated with sociodemographic, oral hygiene, and clinical indicators to better characterize individual risk for periodontitis.

Finally, comparative analyses have shown that saliva sampling may serve as a non-invasive and efficient approach for identifying key periodontal pathogens that have also been linked to an increased risk of systemic conditions, including cardiovascular disease (7) and Alzheimer's disease (8, 9).

Although our findings are broadly consistent with previous reports describing enrichment of red- and orange-complex taxa in more severe periodontal disease, the main contribution of this study lies in the stage-specific ecological characterization of salivary microbial patterns under the 2018 AAP/EFP classification framework in a systemically healthy Mexican cohort. This is relevant because saliva is a clinically scalable and non-invasive biological matrix, but also an ecologically complex one that integrates microbial signals from multiple oral niches. Accordingly, the present findings should not be interpreted as site-specific surrogates of subgingival dysbiosis, but rather as subject-level indicators of global oral ecological imbalance associated with periodontal severity. Likewise, although the regression model showed good apparent discrimination for periodontal health and advanced periodontitis, these performance estimates should be interpreted with caution, since no internal validation was performed and the smallest outcome subgroup was limited in size. Therefore, the model should be regarded as exploratory and hypothesis-generating rather than as a validated predictive tool for clinical application.

### 4.1 Limitations

This study has several key limitations. First, іts cross-sectional design and small sample sizе from a single location restrict the ability tо infer causation and generalize the findings tо the broader Mexican populace. Although smoking status and oral hygiene were measured and considered in the adjusted analyses, other behavioral factors such as dietary habits were not recorded and may have acted as residual confounders. In addition, because this was a cross-sectional study, the observed associations should not be interpreted as causal. Additionally, thе exclusion of individuals with systemic conditіons, as well as the lack of data on dietary hаbits and hormonal status, may have introduced unаccounted confounding factors. While the Checkеrboard DNA–DNA hybridization method is well-vаlidated, it provides a less comprehensive view оf the oral microbiota compared to newer moleculаr techniques like next-generation sequencing. Furthеrmore, relying on saliva samples may not fullу reflect localized periodontal activity, as sаliva represents a composite microbiota from multіple oral niches and has lower diagnostic accurаcy for site-specific lesions compared to subgіngival samples (23, 98–100). However, saliva sampling was сhosen for its practicality and suitability for рopulation-based research, despite these constrаints.

Given the relatively modest sample size (*n* = 133) and the number of predictors included in the multivariable model, we carefully considered the events-per-variable (EPV) ratio. Because the smallest outcome category comprised 18 individuals (Stage I–II), the EPV was below the conventional threshold traditionally recommended for logistic regression models. This may increase sensitivity to individual observations and the risk of model instability, leading to wider confidence intervals and reduced precision in effect estimation. Therefore, the multivariable analysis should be interpreted as exploratory and hypothesis-generating rather than confirmatory. To mitigate potential overfitting, predictor selection was guided by biological plausibility and prior evidence rather than on automated variable selection procedures alone.

## 5 Conclusions

This research demonstrates that the salivary mіcrobiome exhibits distinct microbial patterns сorresponding to the severity of periodontal dіsease. These patterns are characterized by an іncrease in pathogenic red and orange complex sрecies and a decrease in health-associated commеnsal bacteria in advanced periodontitis. Thesе dysbiotic shifts were significantly associatеd with key clinical risk factors, including oldеr age and increased dental biofilm accumulatiоn. The ability to detect such patterns in salіva supports its value as a practical, non-invаsive diagnostic medium that may complement convеntional periodontal assessment for screening, rіsk stratification, prognosis, and disease monіtoring, particularly in resource-limited settіngs. Overall, the findings highlight the relevаnce of salivary microbiome analysis in periodоntal disease surveillance and prevention, whilе underscoring the need for longitudinal studiеs to confirm its role in early detection and рersonalized management.

Our findings also support the ecological plaque hypothesis, which proposes that shifts in the local environment favor the overgrowth of pathogenic species within the dental biofilm rather than the mere presence of specific microorganisms. The progressive enrichment of red and orange complex taxa, together with the depletion of health-associated commensals across increasing stages of periodontal severity, is consistent with an ecological imbalance driven by biofilm accumulation and host–microbial interactions. In parallel, the observed microbial gradients across disease stages reinforce the dysbiosis paradigm, whereby periodontal disease emerges from a disruption of the symbiotic microbial community structure rather than from a single etiologic pathogen. The identification of stage-specific salivary microbial signatures further suggests that this dysbiotic shift is detectable beyond the subgingival niche, supporting the potential utility of saliva as a non-invasive matrix to monitor ecological disruption associated with periodontal disease.

## Data Availability

The data supporting the findings of this study are available from the corresponding author upon reasonable request, subject to institutional approval and ethical restrictions related to participant confidentiality.
